# Supplementary material for: Efficacy and Safety of a Booster Vaccination with Two Inactivated SARS-CoV-2 Vaccines on Symptomatic COVID-19 Infection in Adults: Results of a Double-Blind, Randomized, Placebo-Controlled, Phase 3 Trial in Abu Dhabi
Source: Vaccines (Basel). 2023 Jan 30;11(2):299. doi: 10.3390/vaccines11020299 (PMC9962595; doi:10.3390/vaccines11020299)
Supplement: Supplementary file 1 [file vaccines-11-00299-s001.zip › vaccines-2108764-supplementary.pdf]

Supplement

eMethods

1. Detailed inclusion and exclusion criteria

Participants were considered eligible for the study if they met the following criteria:

- Age range: healthy people aged 18+ years old;
- General good health as established by medical history and physical examination;
- Women of childbearing age are not pregnant (negative urine pregnancy test), are not breastfeeding, do not have pregnancy plan within the three months after enrollment, and have already taken effective contraceptive measures two weeks before enrollment;
- Participants are able and willing to complete the whole research procedure;
- Participants have the ability to understand the research procedures, to sign the informed consent voluntarily after explanation, and can comply with the requirements of the clinical research program.

Those who met the inclusion criteria were further evaluated for the following exclusion criteria before the first dose:

- Confirmed acute cases of SARS-CoV-2 infection;
- With a medical history of SARS, MERS virus infection (self-report, on-site inquiry);
- Fever (axillary temperature > 37.0 ℃), dry cough, fatigue, nasal obstruction, runny nose, pharyngeal pain, myalgia, diarrhea, shortness of breath and dyspnea within 14 days before vaccination (Tympanic temperature / Temporal artery temperature > 37.5 ℃);
- Positive urine pregnancy test result;
- Body temperature axillary ≥ 37.0 ℃ before vaccination（Tympanic temperature / Temporal artery temperature≥ 37.5 ℃);
- With previous severe allergic reactions (such as acute allergic reactions, urticaria, skin eczema, dyspnea, angioneurotic edema or abdominal pain) or allergy to known ingredients of the inactivated SARS-CoV-2 vaccine;
- With a medical history or family history of convulsion, epilepsy, encephalopathy or mental illness; With congenital malformation or developmental disorder, genetic defects, severe malnutrition, etc.;
- With known or suspected diseases include acute respiratory diseases (e.g. influenza like illness, acute cough, sore throat), severe cardiovascular diseases, severe liver diseases, severe kidney diseases, uncontrollable hypertension (systolic blood pressure > 150 mmHg, diastolic blood pressure > 90 mmHg), diabetic complications, malignant tumors, various acute diseases, or acute attack period of chronic diseases.
- Has been diagnosed with congenital or acquired immune deficiency, HIV infection, lymphoma, leukemia or other autoimmune diseases;
- With a history of coagulation dysfunction (such as coagulation factor deficiency, coagulation disease).
- Receiving anti-TB therapy;
- Receiving immune enhancement or inhibitor therapy within 3 months (continuous oral or IV administration for more than 14 days);
- Vaccinated live attenuated vaccine within 1 month before vaccination and other vaccines within 14 days before vaccination;

— Received blood products within 3 months before vaccination;

— Received other investigational drugs within 6 months before vaccination;

— Other circumstances judged by investigators that were not suitable for participating in this clinical trial.

2. The definition and description of the analysis set

(1) Full Analysis Set (FAS)

Includes all subjects who follow intent to treat (ITT) principle, undertake randomization, received the booster dose of vaccine, PCR test is negative during screening and have ≥1 follow up for case surveillance. Subjects needed to be excluded from FAS if other COVID-19 vaccines have been recorded prior to the booster immunization. Subjects who were wrongly vaccinated were randomized to evaluate their protective efficacy according to ITT principles

(2) Modified Full Analysis Set (mFAS)

Includes all subjects who received the booster dose of vaccination except:

— Subjects did not enter the case surveillance period whose vaccine shed within 14 days after booster dose;

— less than 14 days after the booster dose to the cut-off date of data analysis;

—PCR-positive subjects within 14 days of booster dose;

— Subjects diagnosed with COVID-19 within 14 days of booster dose;

— Received other COVID-19 vaccines within 14 days of booster dose;

— PCR positive at the screening period.

(3) Vaccine efficacy Per protocol Set Analysis Set (PPS, Per Protocol Set)

The subjects in PPS are more compliant to the protocol, including all subjects who met the inclusion/exclusion criteria, participate in randomization, received booster dose of vaccination according to the protocol requirements. Among them, subjects who met the following conditions should be excluded from PPS:

— Subjects did not enter the case surveillance period whose vaccine shed within 14 days after booster dose;

— less than 14 days after the booster dose to the cut-off date of data analysis;

—PCR-positive subjects within 14 days of booster dose;

— Subjects diagnosed with COVID-19 within 14 days of booster dose;

— Received other COVID-19 vaccines within 14 days of booster dose;

— The subjects received the wrong vaccination or incorrect dose of vaccine;

— PCR positive at the screening period;

—Use of vaccines or drugs prohibited by the protocol within 14 days of booster dose;

— Other situations that affect the evaluation of the vaccine efficacy

(4) Immunogenicity Full Analysis Set (iFAS)

Includes all subjects who follow intent to treat (ITT) principle, undertake randomization, received the booster dose of vaccine, and collected blood with effective antibody titer value before completion of immunization. Subjects needed to be excluded from FAS if other COVID-19 vaccines have been recorded prior to the booster immunization. Subjects who were wrongly vaccinated were randomized to evaluate their immunogenicity analysis according to ITT principles.

(5) Immunogenicity Per Protocol Set 14 days after booster shot (Immunogenicity Per Protocol Set 1，iPPS1)

Includes all subjects who met the inclusion/exclusion criteria, participate in randomization, received

booster dose of vaccination according to the protocol requirements, collected blood with effective antibody titer value before completion of immunization and 14 days after the booster dose. Subjects who met the following conditions needed to go through a blinded data review meeting to decide whether to enter iPPS1:

— The subjects violated the test protocol before blood collection

— Received other COVID-19 vaccines within 14 days of booster dose;

— The subjects received the wrong vaccination or incorrect dose of vaccine;

—Use of vaccines or drugs prohibited by the protocol within 14 days of booster dose;

— Other situations that affect the evaluation of the immunogenicity analysis.

(6) Immunogenicity Per Protocol Set 28 days after booster shot (Immunogenicity Per Protocol Set 2，iPPS2)

Includes all subjects who met the inclusion/exclusion criteria, participate in randomization, received booster dose of vaccination according to the protocol requirements, collected blood with effective antibody titer value before completion of immunization and 28 days after the booster dose. Subjects who met the following conditions needed to go through a blinded data review meeting to decide whether to enter iPPS2:

— The subjects violated the test protocol before blood collection

— Received other COVID-19 vaccines within 28 days of booster dose;

— The subjects received the wrong vaccination or incorrect dose of vaccine;

—Use of vaccines or drugs prohibited by the protocol within 28 days of booster dose;

— Other situations that affect the evaluation of the immunogenicity analysis.

(7) Safety Set (SS)

Includes all subjects who received the booster dose of vaccine. Among which, subjects with wrong vaccination were conducted safety analysis according to the actual treatment group as per All Subjects as Treated (ASaT) principle.

3. Case Definition

(1) Suspected cases:

Comprehensive judgment based on epidemiological history and clinical symptoms:

— Have any of the epidemiological history, and have two or more A symptoms, or have one or more B symptoms; or with imaging features of COVID-19.

— If there is no clear epidemiological history, they should have two or more A symptoms or one or more B symptoms and detectable SARS-CoV-2 specific IgM; or have two or more A symptoms and One or more B symptoms; or with imaging features of COVID-19.

Epidemiological history:

— Long-term residence or stay in the epidemic area for more than 7 days; or History of travel or residence in the community where the case was reported within 14 days before the onset of illness; or

— In contact with SARS-CoV-2 infected or asymptomatic infected persons within 14 days before the onset of illness; or

— Cluster cases (2 or more cases of fever and/or respiratory symptoms occurred in a small area such as home, office, school, etc. within 2 weeks).

Clinical symptoms:

— Symptoms A (present for at least 2 days): fever (axillary temperature ≥37.5°C); chills; sore throat; fatigue; nasal congestion or runny nose; body pain, muscle pain; headache; nausea or vomiting; diarrhea.

— Symptoms B: Cough (present for at least 2 days); new taste or smell disorders (present for at least 2 days); shortness of breath or difficulty breathing;

Imaging features of COVID-19:

— According to local clinical diagnosis and treatment protocol

(2) Confirmed cases:

— On the basis of the determination of the suspected case, the SARS-COV-2 PCR detection test result is positive.

— Encourage any possible etiological differential diagnosis.

(3) Clinical classification:

Confirmed mild COVID-19 cases:

— The clinical symptoms were mild, and there was no sign of pneumonia on imaging.

Confirmed moderate COVID-19 cases:

— Showing fever and respiratory symptoms with radiological findings of pneumonia.

Confirmed severe COVID-19 cases:

Confirmed COVID-19 case meeting any one of the following criteria:

— Respiratory distress (RR≥30 breaths/min);

— Oxygen saturation≤93% at rest;

— Arterial partial pressure of oxygen (PaO2)/ fraction of inspired oxygen (FiO2)≦300mmHg (1mmHg=0.133kPa);

— The clinical symptoms progressively worsened, and the chest imaging showed >50% obvious lesion progression within 24-48 hours.

Confirmed Critical COVID-19 cases:

Confirmed COVID-19 case meeting any one of the following criteria:

— Respiratory failure and requiring mechanical ventilation;

— Shock;

— Other organ failure that requires ICU care;

— Death

4. Method for infectious SARS-Cov-2 neutralizing assay

The neutralization antibody assays were performed at the National Institute of Viral Disease Control and Prevention, Beijing, China. Serum was successively diluted 1:4 to the required concentration by a 2-fold series, and an equal volume of challenge virus solution was added. After neutralization in a 37 °C incubator for 2 h, a $1.0\sim2.5\times10^5$/ml cell suspension was added to the wells (0.1 ml/well) and cultured in a CO2 incubator at 37 °C for 4 days. Titers expressed as the reciprocal of the highest dilution protecting 50% cell from virus challenge. Convalescent sera is included as an internal positive control in every assay. Seroconversion was defined as an increase in post-vaccination titer of four-fold or more from baseline.

eTables

eTable S1. Efficacy After 14 Days Following booster Dose: Subgroup Analysis

Sensitivity analysis	WIV04 vaccine group	HB02 vaccine group	Alum-only group
**Modified Full Analysis Set**			
No. of participants	3006	3055	3010
No. of incident cases	36	28	193
Incidence density per 1000 person-years (95% CI)	1.2 (0.8, 1.7)	0.9 (0.6, 1.3)	6.4 (5.6, 7.4)
Vaccine efficacy (95% CI), %	81.3 (73.4, 86.9)	85.7 (78.8, 90.4)	Reference
**Per protocol Set Analysis Set**			
No. of participants	3005	3054	3008
No. of incident cases	36	28	193
Incidence density per 1000 person-years (95% CI)	1.2 (0.8, 1.7)	0.9 (0.6, 1.3)	6.4 (5.6, 7.4)
Vaccine efficacy (95% CI), %	81.3 (73.5, 86.9)	85.7 (78.8, 90.4)	Reference

Abbreviation: alum, aluminum hydroxide; WIV04 and HB02 groups represent the vaccine groups which were developed based on two SARS-CoV-2 strains, WIV04 and HB02 strains.Per protocol Set Analysis Set was a subset of Modified Full Analysis Set in the main analysis, Incidence density with its 95% CI was estimated using the least-square method.

eTable S2. Efficacy After 14 Days Following booster Dose: Subgroup Analysis

**Subgroup**	**WIV04**	**HB02**	**Alum only**
**Female**			
No. of participants	466	455	425
No. of incident cases	7	3	29
Person-years	53.0	53.1	47.3
Incidence density, per 1000 person-years (95% CI)	13.2(6.3, 27.7)	5.7(1.8, 17.5)	61.3(42.6, 88.2)
Vaccine efficacy, % (95% CI)	82.7(74.2, 88.8)	85.5(77.8, 90.9)	Reference
**Male**			
No. of participants	2540	2600	2585
No. of incident cases	29	25	164
Person-years	309.3	317.3	302.5
Incidence density, per 1000 person-years (95% CI)	9.4(6.5, 13.5)	7.9(5.3, 11.7)	54.2(46.5, 63.2)
Vaccine efficacy, % (95% CI)	78.4(49.7, 92.0)	90.8(70.3, 98.2)	Reference
**Subgroup**	**WIV04**	**HB02**	**Alum only**
**<60 years**			
No. of participants	2891	2956	2886
No. of incident cases	35	27	190
Person-years	352.0	360.5	337.9
Incidence density, per 1000 person-years (95% CI)	9.9 (7.1, 13.9)	7.5 (5.1, 10.9)	56.2 (48.8, 64.8)
Vaccine efficacy, % (95% CI)	82.3(74.5, 88.0)	86.7 (80.0, 91.5)	Reference
**Subgroup**	**WIV04**	**HB02**	**Alum only**
**⩾60 years**			
No. of participants	115	99	124
No. of incident cases	1	1	3
Person-years	10.3	9.9	11.9
Incidence density, per 1000 person-years (95% CI)	9.7 (1.4，69.1)	10.1 (1.4，71.9)	25.3 (8.2，78.4)
Vaccine efficacy, % (95% CI)	61.5 (-379.5，99.3)	59.9 (-399.1，99.2)	Reference

Abbreviation: alum, aluminum hydroxide; WIV04 and HB02 groups represent the vaccine groups which were developed based on two SARS-CoV-2 strains, WIV04 and HB02 strains.

A Poisson regression model with log link function was employed with the number of incident cases as the dependent variable, treatment group as the independent variable and the person-years as offset. Incidence density with its 95% CI was estimated using the least-square method. If the number of cases in any of the groups was <5, the exact method was used to estimate the incidence rate, vaccine efficacy and 95% CI by using StatXact software.

eTable S3. Neutralizing Antibodies to Live SARS-CoV-2 for Male/Female Group

14 d after the booster dose-male	WIV04 vaccine group (n=252)	HB02 vaccine group (n=259)	Alum-only group (n=246)	P-value
N(Missing)	252 (0)	259 (0)	246 (0)	
Geometric mean titers[a]	203.1	493.1	4.7	<0.0001
95%CI[b]	174.0, 237.1	441.8, 550.4	4.0, 5.5	
**14 d after the booster dose-female**	**WIV04 vaccine group (n=58)**	**HB02 vaccine group (n=41)**	**Alum-only group (n=44)**	**P-value**
N(Missing)	58 (0)	41 (0)	44 (0)	
Geometric mean titers	152.0	484.8	3.2	<0.0001
95%CI	105.8, 218.4	365.7, 642.8	2.3, 4.4	
**28 d after the booster dose-male**	**WIV04 vaccine group (n=252)**	**HB02 vaccine group (n=259)**	**Alum-only group (n=246)**	**P-value**
N(Missing)	252 (0)	259 (0)	246 (0)	
Geometric mean titers	299.5	822.4	5.4	<0.0001
95%CI	255.9, 350.6	727.0, 930.4	4.7, 6.4	
**28d after the booster dose-female**	**WIV04 vaccine group (n=58)**	**HB02 vaccine group (n=41)**	**Alum-only group (n=44)**	**P-value**
N(Missing)	58 (0)	41 (0)	44 (0)	
Geometric mean titers	212.2	762.1	3.5	<0.0001
95%CI	148.3, 303.6	563.4, 1031.0	2.4, 4.9	

a. the main analyses were performed after log transformation and results are expressed in terms of geometric mean

b. The 95% confidence intervals (CI) were calculated using the exact binomial (Clopper-Pearson) methodology.

eTable S4.Seropositive rate against 10 ancestral strains

Virus strain	Seropositive rate	WIV04 group (n=50)	HB02 group (n=47)	Alum only group (n=53)
2366T	⩾4 n (%)	50 (100.0)	47 (100.0)	8 (15.1)
	95% CI	92.9, 100.0	92.5, 100.0	6.8, 27.6
77	⩾4 n(%)	50 (100.0)	47 (100.0)	7 (13.2)
	95% CI	92.9, 100.0	92.5, 100.0	5.5, 25.3
76	⩾4 n(%)	50 (100.0)	47 (100.0)	4 (7.6)
	95% CI	92.9, 100.0	92.5, 100.0	2.1, 18.2
F13 P4	⩾4 n(%)	50 (100.0)	47 (100.0)	7 (13.2)
	95% CI	92.9, 100.0	92.5, 100.0	5.5, 25.3
35T P2	⩾4 n(%)	50 (100.0)	47 (100.0)	6 (11.3)
	95% CI	92.9, 100.0	92.5, 100.0	4.3, 23.0
56Y P3	⩾4 n(%)	50 (100.0)	47 (100.0)	7 (13.2)
	95% CI	92.9, 100.0	92.5, 100.0	5.5, 25.3
HN97 P3	⩾4 n(%)	50 (100.0)	47 (100.0)	7 (13.2)
	95% CI	92.9, 100.0	92.5, 100.0	5.5, 25.3
834 Y	⩾4 n(%)	50 (100.0)	47 (100.0)	6 (11.3)
	95% CI	92.9, 100.0	92.5, 100.0	4.3, 23.0
QD01	⩾4 n(%)	50 (100.0)	47 (100.0)	8 (15.1)
	95% CI	92.9, 100.0	92.5, 100.0	6.8, 27.6
P701	⩾4 n(%)	50 (100.0)	47 (100.0)	5 (9.4)
	95% CI	92.9, 100.0	92.5, 100.0	3.1, 20.7

Abbreviation: alum, aluminum hydroxide; WIV04 and HB02 groups represent the vaccine groups which were developed based on two SARS-CoV-2 strains, WIV04 and HB02 strains.

WIV04：hCoV-19/Wuhan/WIV04/2019; HB02：19nCoV-CDC-Tan-HB02

2366T: BetaCoV/Qingdao/IVDC-BJ-Xinfadi2366T/2020; 77: BetaCoV/Qingdao/IVDC-QD-envCodPackage77 /2020;

76: BetaCoV/Qingdao/IVDC-QD-Philippines76/2020; F13 P4: BetaCoV/Wuhan/IVDC-HB-envF13/2020;

35T P2: BetaCoV/Beijing/IVDC-BJ-uk35T/2020: 56Y P3: BetaCoV/Beijing/IVDC-BJ-austria56Y/2020:

HN97 P3: BetaCoV/Changde/IVDC-BJ-HN97/2020: 834 Y: BetaCoV/Harbin/IVDC-BJ-russia834BY/2020:

QD01: 19nCoV-CDC-Tan-Strain04（QD01）; P701: W2020（COVID-19-BJ-P7)01.

eTable S5. Vaccine related adverse Reactions after booster Dose in the Safety Set

Adverse event	WIV04 (n=3083)	HB02 (n=3150)	Alum only (n=3076)
**0-7d**			
Total adverse events	1014 (32.9)	896 (28.4)	966 (31.4)
Solicited adverse events	977 (31.7)	850 (27.0)	924 (30.0)
Local events	736 (23.9)	556 (17.7)	672 (21.9)
Pain	718 (23.3)	543 (17.2)	656 (21.3)
Induration	9 (0.3)	4 (0.1)	5 (0.2)
Swelling	26 (0.8)	19 (0.6)	13 (0.4)
Rash	4 (0.1)	8 (0.3)	6 (0.2)
Redness	17 (0.6)	13 (0.4)	9 (0.3)
Itching	17 (0.6)	15 (0.5)	8 (0.3)
Systemic events	426 (13.8)	489 (15.5)	411 (13.4)
Fever	50 (1.6)	49 (1.6)	40 (1.3)
Diarrhea	30 (1.0)	31 (1.0)	24 (0.8)
Constipation	8 (0.3)	9 (0.3)	10 (0.3)
Dysphagia	0 (0.0)	1 (0.0)	6 (0.2)
Anorexia	1 (0.0)	2 (0.1)	2 (0.1)
Vomiting	4 (0.1)	3 (0.1)	2 (0.1)
Nausea	14 (0.5)	22 (0.7)	14 (0.5)
Myalgia (Non-inoculated site)	90 (2.9)	104 (3.3)	95 (3.1)
Arthralgia	11 (0.4)	33 (1.1)	17 (0.6)
Headache	202 (6.6)	243 (7.7)	204 (6.6)
Coughing	44 (1.4)	55 (1.8)	42 (1.4)
Dyspnea	16 (0.5)	14 (0.4)	14 (0.5)
Pruritus (non-inoculated site)	12 (0.4)	20 (0.6)	6 (0.2)

Skin and mucosal abnormalities	2 (0.1)	2 (0.1)	0 (0.0)
Acute allergic reactions	5 (0.2)	5 (0.2)	1 (0.0)
Fatigue	128 (4.2)	165 (5.2)	127 (4.1)
Unsolicited adverse reactions	147 (4.8)	185 (5.9)	157 (5.1)
**8-28d**			
Total adverse reactions	17 (0.6)	18 (0.6)	26 (0.9)
Solicited adverse reactions	0 (0.0)	0 (0.0)	0 (0.0)
Local reactions	0 (0.0)	0 (0.0)	0 (0.0)
Systemic reactions	0 (0.0)	0 (0.0)	0 (0.0)
Unsolicited adverse reactions	17 (0.6)	18 (0.6)	26 (0.9)
**Until 17 October, 2021**			
Total adverse reactions	1023 (33.2)	909 (28.9)	984 (32.0)
Solicited adverse reactions	977 (31.7)	850 (27.0)	924 (30.0)
Local reactions	736 (23.9)	556 (17.7)	672 (21.9)
Systemic reactions	426 (13.8)	489 (15.5)	411 (13.4)
Unsolicited adverse reactions	163 (5.3)	202 (6.4)	181 (5.9)

Abbreviation: alum, aluminum hydroxide. WIV04 and HB02 groups represent the vaccine groups which were developed based on two SARS-CoV-2 strains, WIV04 and HB02 strains.

Data are shown as No. of participants with event (%). A participant was only counted once in the specific reaction category even though a participant could have more than 1 adverse reaction. For example, a participant who had the same symptom (eg, injection site pain) after each dose was counted once in the symptom category. Similarly, if a participant had more than 1 symptom in the reaction class (total, systemic, and local), they were only counted once in that adverse reaction class.

eTable S6. The Number of Serious Adverse Events (SAEs) in the Safety Set

SAEs	WIV04 (n=3083)	HB02 (n=3150)	Alum only (n=3076)
Total SAEs	17 (0.6)	11 (0.4)	40 (1.3)
Certain infectious and parasitic SAEs	7 (0.2)	9 (0.3)	38 (1.2)
SAEs of the nervous system	2 (0.1)	1 (0.0)	1 (0.0)
Injury, poisoning and certain other consequences of external causes	2 (0.1)	0 (0.0)	2 (0.1)
Nutritional and metabolic SAEs	1 (0.0)	0 (0.0)	2 (0.1)
SAEs of kidney and urinary system	1 (0.0)	1 (0.0)	1 (0.0)
SAEs of the gastrointestinal system	2 (0.1)	0 (0.0)	1 (0.0)
Examinations	2 (0.1)	0 (0.0)	0 (0.0)
SAEs of the respiratory system	2 (0.1)	0 (0.0)	0 (0.0)
Mental and behavioral disorders	1 (0.0)	1 (0.0)	0 (0.0)
SAEs of the ear and adnexa	1 (0.0)	0 (0.0)	0 (0.0)
SAEs of the musculoskeletal system and connective tissue	0 (0.0)	1 (0.0)	0 (0.0)
Surgeries and medical procedures	1 (0.0)	0 (0.0)	0 (0.0)
Systemic or injection-site SAEs	1 (0.0)	0 (0.0)	0 (0.0)
SAEs of the reproductive system and breast	0 (0.0)	0 (0.0)	1 (0.0)
SAEs of vessels and lymphatic vessels	0 (0.0)	0 (0.0)	1 (0.0)
SAEs Related to Vaccination	0 (0.0)	0 (0.0)	0 (0.0)
SAEs Not Related to Vaccination	17 (0.6)	11 (0.4)	40 (1.3)

Abbreviation: alum, aluminum hydroxide. WIV04 and HB02 groups represent the vaccine groups which were developed based on two SARS-CoV-2 strains, WIV04 and HB02 strains.

eTable S7. Incident COVID-19 Cases and Vaccine Efficacy at different stages after Second Doses of Immunization

Outcome (median follow-up time, day)	WIV04 vaccine group	HB02 vaccine group	Alum-only group
**Interim analysis (50)**			
No. of participants	13771	13765	13765
No. of incident cases	26	21	95
Person-years	2174.5	2178.0	2161.0
Incidence density per 1000 person-years (95% CI)	1.20(0.81, 1.76)	0.96(0.63, 1.48)	4.40(3.60, 5.38)
Vaccine efficacy (95% CI), %	72.80(58.03, 82.37)	78.07(64.82, 86.33)	
**Final analysis (154)**			
No. of participants	14065	14079	14079
No. of incident cases	139	118	319
Person-years	4027.9	4037.0	4001.0
Incidence density per 1000 person-years (95% CI)	3.45(2.92, 4.08)	2.92(2.44, 3.50)	7.97(7.14, 8.90)
Vaccine efficacy (95% CI), %	56.72(47.18, 64.53)	63.34(54.72, 70.32)	
**Protection persistence analysis (213)**			
No. of participants	14156	14175	14169
No. of incident cases	291	260	594
Person-years	6046.2	6068.4	5978.9
Incidence density per 1000 person-years (95% CI)	4.81(4.29, 5.40)	4.28(3.79, 4.84)	9.93(9.17, 10.77)
Vaccine efficacy (95% CI), %	51.55(44.26, 57.89)	56.87(50.11, 62.72)	
